# Special Hospitals and Graduate Study

**Published:** 1920-09-04

**Authors:** 


### Special Hospitals and Graduate Study

**Central London Throat and Ear Hospital, Gray's Inn Road, W.C.**—Three courses of instruction are open to practitioners attending the hospital: first, the course in methods of examination and diagnosis; second, the course of systematic instruction in the diseases of the nose, throat, and ear; and third, the operative surgery class. The course in methods of examination and diagnosis is introductory in character. It comprises four lessons of practical teaching in the actual examination of patients and in the manipulation of instruments. Clinical assistants, especially if they have not had any previous experience in the speciality, are expected to attend this class in order to become acquainted with the minutiæ of the methods of diagnosis, etc. The class is free to clinical assistants. To ordinary students and practitioners the fee is one guinea. Intending students (who can join at any time) are requested to give their names to the Dean. Attendance at this class is compulsory for those who desire to obtain the higher-grade certificate. The second course of systematic instruction in diseases is more advanced. It consists of over thirty lessons in all on pathology, diagnosis, and treatment. Full syllabus may be had on application to the Secretary.

**Hospital for Consumption and Diseases of the Chest, Brompton, S.W.**—Students may attend this hospital at a fee of £2 2s. for one month, £4 4s. for three months, or £7 7s. for six months. Demonstrations are given in the wards, out-patient department, *x*-ray, and throat departments.

Clinical assistants are appointed to the physicians and assistant physicians.

For particulars apply to the Dean, Dr. L. S. Burrell.

**Hospital for Diseases of the Throat, Golden Square, London, W.,** with which is amalgamated the London Throat Hospital, Great Portland Street.—The hospital contains sixty beds. There is an out-patient attendance of over 60,000 yearly. Clinical instruction in the diagnosis and treatment of disease is given in the out-patient department from 2 to 5 P.M. on Tuesdays, and Fridays from 6.30 to 9 P.M. From amongst the students junior clinical assistants are selected, whose duty it is to assist the member of the staff to whom they are appointed. For terms and further information apply to the Dean, Mr. Lionel Colledge.

**Hospital for Sick Children, Great Ormond Street, W.C.**—This school is one of the post-graduate teaching schools of the University of London, and is recognised by the Conjoint Board of England and by the Universities of London, Oxford, and Cambridge, as a place where undergraduates may spend six months of their fifth year in clinical work, or hold the appointments of clinical clerks and dressers. There are 240 beds, to

(*Continued on p.* xvi.)

which nearly 3,000 cases are admitted per annum, and in the out-patient department the average number of new cases each year exceeds 25,000. Clinical instruction is given daily in the wards, out-patient department, and operating theatre by members of the visiting staff. Fees for one month's attendance, two guineas; for three months, five guineas; perpetual ticket, ten guineas; clinical clerk's reduced fee of £1 1s. for one month. A certificate is given at the end of a three months' course. Further details and full syllabus may be obtained by application to the Dean, Sub-Dean, or the Secretary of the hospital. A limited number of clerkships in the pathological department, affording facilities for theoretical and practical instruction in clinical pathology and bacteriology suitable for post-graduates and fifth-year students, are available. Fees for one month's course, three guineas; two months' course, five guineas; three months' course, six guineas. For detailed syllabus application should be made to the Dean or the Secretary. The Dean is Mr. O. L. Addison and the Sub-Dean Dr. W. J. Pearson, from whom all further information can be obtained, or from the Secretary at the hospital.

**The Hospital for Women, Soho Square, W.**—In connection with the out-patient department there has been for some years a well-organised clinical department. To meet the want increasingly felt by medical practitioners of a more intimate knowledge of the diseases peculiar to women, and of more extended opportunities for examining gynæcological cases, clinical assistants are appointed to the gynæcologists to out-patients. The appointments are open to qualified medical men and women.

The hospital contains sixty-eight beds. In the out-patient department 4,000 cases are treated every year, the total number of out-patient attendances being from 14,000 to 15,000. This large number affords exceptional opportunities for seeing and studying most of the varieties of the diseases of women. Clinical assistants are entitled to receive notice of all operations performed within the hospital upon giving their names and addresses to the hall porter, and every facility is afforded them by the gynæcologists in the out-patient department of examining patients and obtaining experience in diagnosis and treatment.

The number of clinical assistants appointed to each gynæcologist is, so far as possible, limited to two, though occasionally a third may be appointed. The clinical assistants attend on two afternoons a week, and it is advisable that they should take out a three months' course, but to meet the convenience of medical practitioners it is permitted to attend for a shorter period, and on one or more days a week if it can be arranged. A fee is charged at the rate of two guineas a month, for two days a week, which becomes due on joining. A certificate is given at the end of a three months' course.

The out-patients are seen daily at 1.30 P.M., except on Saturdays, when they are seen at 9.30 A.M. Any further information can be obtained by writing to the Secretary at the hospital.

**National Hospital for the Paralysed and Epileptic, Queen Square, W.C.**—The in- and out-patient practice of this hospital is open at 2 P.M. every day (except Wednesday and Saturday). Patients are seen by the physicians, the physicians for out-patients, and the assistant physicians. Three courses of lectures and clinical demonstrations are given in each year. The fee for out-patient hospital practice and lectures is five guineas for three months, seven guineas for six months, and ten guineas for a perpetual ticket; for in-patient clerking a fee of five guineas for three months or seven guineas for six months is charged. The museum is available for the prosecution of special work under the pathologist, for which a separate fee is charged. Women students attend the practice of the hospital.

All communications should be sent to the Dean of the Medical School.

**Ophthalmic Hospitals.**—Ophthalmic hospitals are the Central London Ophthalmic Hospital, Judd Street, W.C.; the Royal London Ophthalmic Hospital, City Road, E.C.; the Royal Eye Hospital, St. George's Circus, S.E.; and the Royal Westminster Ophthalmic Hospital, King William Street, W.C. In each of these the fees charged are very modest.

**Queen Charlotte's Lying-in Hospital.**—Qualified medical practitioners and medical students are admitted to the practice of this hospital. Last year (1919) there were 1,991 women delivered in the wards, about one-half being primiparæ. A large number of the cases were abnormal. During the present year the number of women delivered in the wards has been at the rate of over 2,000 per annum. In the ante-natal department 5,088 patients attended for examination and treatment. Certificates of attendance at this hospital are recognised by all Universities, Colleges, and licensing bodies. Fee for the course of four weeks, £8 8s. Students are accommodated at the new Residential College (5 Cosway Street), opposite the hospital.

This instruction will include: (1) Practical instruction in the methods of examination of pregnant women; (2) delivery of women in labour under the direct supervision of a medical officer of the hospital; (3) practical instruction in the treatment of the mother and child during the puerperium, including clinics held four times weekly by the visiting medical staff; (4) instruction in the clinical laboratory of the hospital.

Sixty-six students (forty-four men and twenty-two women) and ninety-nine qualified practitioners (ninety men and nine women) attended the practice of the hospital during 1919.

**Royal Hospital for Diseases of the Chest, City Road, E.C.**—This hospital, which has lately undergone extensive reorganisation, provides an excellent field for research and study in diseases of the chest, more especially in tuberculosis in its various departments. The departments in which study can be pursued comprise the in-patient, out-patient, Röntgen-ray, cardiological, pathological and bacteriological, ear, nose and throat, and dental departments. An extensive department for the prevention of consumption (tuberculosis dispensary) has been established for nearly four years. As this works in connection with three large boroughs—namely, Shoreditch, Finsbury, and Islington—a splendid opportunity presents itself for medical practitioners and students who desire to obtain an intimate knowledge of this work. The bacteriological and pathological research laboratories, under the new director, Dr. Carnegie Dickson, should provide excellent opportunities for acquiring a knowledge of bacteriological methods, and also a field for research for those who wish to pursue such studies. The cardiological department, which has recently been established, has been equipped with all the latest apparatus for the investigation of diseases of the heart. In connection with the Röntgen-ray department, there are two radiologists, who attend the hospital four days weekly. Attached to the hospital is a medical school for instruction in diseases of the chest, more especially tuberculosis, at which courses of instruction are given from time to time. There is also

(*Continued on p.* xix.

a tuberculosis school for nurses, where regular theoretical and thoroughly practical courses of instruction are given, for which a certificate is granted. The fees for attending the clinical practice of the hospital are as follows: One month, one guinea; three months, three guineas; six months, five guineas; perpetual ticket, six guineas. Any further information will be gladly given by the Dean, Major Barty King.

**St. John's Hospital for Diseases of the Skin.**—Out-patient Department, 49 Leicester Square, W.C. 2; In-patient Department, 262 Uxbridge Road, W. This hospital has a well-equipped in-patient department, with 40 beds. It has a School of Dermatology at 49 Leicester Square, which is conducted by the medical staff of the hospital. During the winter the free course of Chesterfield lectures is given on Thursday evenings at six o'clock by Drs. Wm. Griffith, J. L. Bunch, and Knowsley Sibley, and is always well attended by the profession. The opening lecture will be given by Dr. Griffith on Thursday, October 14. The out-patient department contains a spacious laboratory and an electrical department. Special courses in the pathology and bacteriology of the skin may be arranged for. The daily clinics at 2, and every evening (except Saturday) at 6, are open to practitioners upon signing the Visitors' Book. Venereal diseases, under the Government scheme, are seen at all clinics.

### TEACHING INSTITUTIONS IN DENTISTRY.

BESIDES those centres mentioned on other pages, there are in London dental schools connected with Guy's Hospital, the London Hospital, University College Hospital, and the Royal Free Hospital School of Medicine for Women. In each case the equipment is excellent, and a complete curriculum is provided in all subjects for the L.D.S. diploma. Provincially must be mentioned the Birmingham Dental Hospital, Leeds University School of Dentistry, the Dental Hospital of Manchester, Newcastle-upon-Tyne Dental Hospital and School, and the Devon and Exeter Dental Hospital. In Edinburgh the Dental Hospital and School is located in a spacious and well-equipped building at 31 Chambers Street, and offers special advantages to dental students. At Glasgow Dental Hospital the school is open to men and women, and the Glasgow Royal Infirmary has attached to it an excellent dental department. The later stages of training in dentistry are provided in Ireland at the Dental Hospital, Lincoln Place, Dublin.

